# Nerandomilast attenuates idiopathic inflammatory myopathy-associated interstitial lung disease via inhibiting proliferation and differentiation of B cells

**DOI:** 10.3389/fimmu.2026.1771007

**Published:** 2026-02-18

**Authors:** Yuming Liu, Yayue Hu, Se-rigeleng, Zhongyi Yang, Xueze Liu, Jiayang Yu, Huihui Li, Songtao Gu, Weitao Yang, Cheng Yang, Honggang Zhou, Yujie He, Chunyu Kong, Xiaoting Gu, Xiaohe Li

**Affiliations:** 1 State Key Laboratory of Medicinal Chemical Biology, College of Pharmacy, Tianjin First Central Hospital, Nankai University, Tianjin, China; 2 Tianjin Key Laboratory of Molecular Drug Research, Tianjin International Joint Academy of Biomedicine, Tianjin, China; 3 Department of Respiratory & Critical Care Medicine, Tianjin Chest Hospital, Tianjin, China; 4 Department of Rheumatology and Immunology, Xuchang Central Hospital, Xuchang, Henan, China; 5 Department of Rheumatology and Immunology, The First Affiliated Hospital of Zhengzhou University, Zhengzhou, Henan, China

**Keywords:** B cells, cAMP, IIM-ILD, Nerandomilast, PDE4B

## Abstract

**Background:**

Idiopathic inflammatory myopathy-associated interstitial lung disease (IIM-ILD) is a severe autoimmune condition with limited treatment options. Phosphodiesterase 4B (PDE4B) is a key enzyme in the metabolism of cyclic adenosine monophosphate (cAMP) in lung tissue, and targeting PDE4B has been proposed as a promising therapeutic strategy. This study aimed to evaluate the therapeutic potential of Nerandomilast (a PDE4B inhibitor) in an experimental IIM-ILD model and to investigate its underlying mechanisms.

**Methods:**

An IIM-ILD mouse model was established by immunization with skeletal muscle homogenate. Mice were treated with Nerandomilast (5 or 12.5 mg/kg, twice daily) or Nintedanib (60 mg/kg, once daily) as a positive control. Disease severity was assessed using myositis scores and spleen index. Pulmonary fibrosis and inflammation were evaluated via micro-CT, histopathology, and bronchoalveolar lavage fluid (BALF) analysis. B cell infiltration, activation, and differentiation were examined by flow cytometry, immunofluorescence, and Western blotting. Key signaling pathways were analyzed in lung tissue.

**Results:**

Nerandomilast ameliorated muscle inflammation, pulmonary fibrosis, and pulmonary inflammation. Mechanistically, Nerandomilast targeted lung-infiltrating B cells: it inhibited their accumulation and proliferation, downregulated the activation marker BAFF, and suppressed their differentiation into plasma cells by reducing the expression of key transcription factors and the plasma cell marker. Serological testing indicated a significant decrease in anti-Jo-1 autoantibody positivity. At the molecular level, Nerandomilast elevated lung tissue cAMP levels, inhibited the phosphorylation of pro-survival/activation pathways (PI3K/AKT, NF-κB, STAT3) in B cells, and enhanced CREB phosphorylation.

**Conclusion:**

The PDE4B inhibitor Nerandomilast demonstrates potent therapeutic effects in a preclinical IIM-ILD model, alleviating both myositis and pulmonary pathology. Its efficacy is mechanistically linked to the direct modulation of B cells, achieved by elevating intracellular cAMP and subsequently reprogramming key signaling networks to inhibit B cell activation, proliferation, and pathogenic differentiation into antibody-producing plasma cells. These findings highlight Nerandomilast as a promising candidate for the treatment of IIM-ILD.

## Introduction

Idiopathic Inflammatory Myopathy (IIM) is an autoimmune disorder which is typified by persistent muscle inflammation and multi-organ pathology. A major complication of IIM is interstitial lung disease (ILD), which is not only highly prevalent but also the leading contributor to mortality in this patient population (1, 2). While the exact pathogenesis of IIM-ILD is not completely understood, current evidence points to a multi-step process. This process is primarily driven by immune dysregulation, which triggers pulmonary inflammation and ultimately leads to fibrotic remodeling. Current treatment, as conditionally recommended in the 2023 ACR/CHEST guidelines, includes mycophenolate mofetil (MMF) among other immunomodulators (3–6). However, existing therapies-including corticosteroids, antifibrotics, and targeted agents-are often limited by suboptimal efficacy, significant side effects, or inadequate response in rapidly progressive disease. Consequently, the development of novel therapies that can precisely target the specific pathological mechanisms of IIM-ILD is urgently required.

In recent years, the role of B lymphocytes in the treatment of IIM-ILD has garnered increasing attention, attributed to the central role of immune dysregulation in autoimmune-related diseases. Although macrophages and activated fibroblasts are direct effector cells of pulmonary fibrosis, broad-spectrum anti-inflammatory or anti-fibrotic therapies targeting them (e.g., corticosteroids, nintedanib) have limited efficacy and significant side effects, highlighting the need for more immunospecific strategies. Despite the involvement of multiple immune cell subsets, robust evidence suggests that B lymphocytes are a more critical driver in IIM-ILD. This perspective is supported by evidence of alterations in B lymphocyte counts and subpopulation distribution in patients, as well as the detection of myositis-specific autoantibodies (MSAs). A retrospective study involving 333 IIM-ILD patients demonstrated that patients positive for anti-MDA5 antibodies exhibited elevated B-cell levels, accompanied by accelerated ILD progression and increased risk of death (7). MSAs play a pivotal role across different IIM subtypes (8). Among MSAs, anti-Jo-1 antibody shows a particularly strong and independent association with ILD (9). These studies have established B lymphocytes and related antibodies as important diagnostic biomarkers and therapeutic targets for IIM-ILD. B cells not only directly cause tissue damage by producing myositis-specific autoantibodies but also function as efficient antigen-presenting cells to activate T cells and secrete various pro-inflammatory (e.g., TNF-α, IL-6) and pro-fibrotic cytokines, thereby simultaneously driving autoimmunity, inflammation, and fibrosis. This multifunctionality positions B cells as a “hub” connecting adaptive immunity with end-stage organ damage. Rituximab (RTX) specifically targets and eliminates B cells by binding to the CD20 antigen. Clinical trials have demonstrated that RTX is effective in over 50% of patients with connective tissue disease-associated interstitial lung disease (CTD-ILD), with a response rate of nearly 50% in the IIM-ILD subtype, surpassing other CTD-ILD subtypes (10). Our team’s preclinical studies showed that the Bruton’s tyrosine kinase (BTK) inhibitor zanubrutinib significantly improved disease manifestations in the IIM-ILD model (11). Collectively, these findings indicate that alterations in the quantity and functional regulation of B lymphocytes influence the progression of IIM-ILD. Therefore, reducing specific antibody levels by eliminating B cells, plasma blasts, and plasma cells offers a promising therapeutic approach for IIM-ILD.

Nerandomilast, a novel phosphodiesterase 4B (PDE4B) inhibitor, has demonstrated significant efficacy in clinical trials for idiopathic pulmonary fibrosis (IPF) and progressive fibrotic interstitial lung disease (12, 13). PDE4B plays a key role in regulating intracellular cAMP level. Preclinical studies have further confirmed that in various pulmonary fibrosis models, including systemic sclerosis-associated ILD (SSc-ILD), silicosis, and IPF, Nerandomilast can increase tissue cAMP levels and exert potent anti-inflammatory and anti-fibrotic effects (14–18). However, the therapeutic efficacy of Nerandomilast in IIM-ILD and its underlying mechanisms remain unexplored. PDE4B is widely expressed in immune cells, and serves to dampen both inflammatory cytokine production and immune cell proliferation and activation. Notably, studies have demonstrated that inhibiting PDE4B can suppress B cell function (19, 20), positioning PDE4B as a “master switch” that controls B cell activity by modulating intracellular cAMP levels. Given the established antifibrotic effects of PDE4 inhibitors and the increasingly prominent role of B cells in IIM-ILD, we propose a novel scientific question focusing on B cells: Does the PDE4B inhibitor Nerandomilast have an ameliorating effect on IIM-ILD, and is its potential efficacy related to directly reshaping the abnormal activation state of B cells? This study aims to validate this hypothesis, seeking to fill the gap in mechanistic understanding between “PDE4-targeting” and “B cell-targeting” therapies. It should be noted that although this animal model recapitulates the core features of human IIM-ILD (e.g., myositis and pulmonary fibrosis), it may not fully reflect disease heterogeneity, and the findings should be considered as preclinical proof-of-concept.

## Experimental materials and methods

### Materials

Professor Feng Sang kindly supplied Nerandomilast (BI 1015550), and the synthesis method has been recorded in a prior published article (15).

### Laboratory animals

We purchased 6–8 weeks female Balb/c wild-type mice and 6–8 weeks male Sprague-Dawley (SD) rats from Beijing Vital River Laboratory Animal Technology. Experiments complied with relevant institutional and national guidelines.

### IIM-ILD model

SD rats were euthanized by intraperitoneal injection of 100 mg/kg pentobarbital sodium, and hind limb skeletal muscles were harvested. Muscle tissues were cut, ground, and homogenized, followed by filtration and centrifugation to obtain a protein solution (15 mg/mL). An equal volume of Complete Freund’s Adjuvant (CFA; Sigma-Aldrich, F5881) was added to the solution and thoroughly emulsified to form a stable emulsion. Mice were immunized via weekly subcutaneous injection of 0.5 mL emulsion into the posterior neck. Pertussis toxin (2 μg; KKL Med, KM10754) was administered intraperitoneally on day 0 and day 7. The drug treatment was administered during the 4th to 5th week after immunization. Subsequently, all mice were anesthetized via intraperitoneal injection of 60 mg/kg pentobarbital sodium, followed by the measurement of pulmonary function parameters and sample collection.

Twenty-five mice were assigned to five experimental groups (n = 5 per group): (1) Control, (2) Model, (3) Nintedanib (60 mg/kg, once daily), (4) Nerandomilast low-dose (5 mg/kg, twice daily), and (5) Nerandomilast high-dose (12.5 mg/kg, twice daily). Nintedanib (Shanghai Macklin Co., Ltd.) was used as a positive control, and the dosage regimens for Nintedanib and Nerandomilast were selected based on previously validated protocols. The sample size (n=5 per group) was chosen based on our laboratory’s prior experience with this model, where it has provided sufficient power to detect significant differences in primary endpoints such as fibrosis area and key inflammatory markers.

### IIM macroscopic pathological evaluation

Subsequent to model induction, the body weight of the mice was measured at a consistent time point on a weekly basis to evaluate their fundamental survival condition. In parallel, the disease status of the mice was recorded and evaluated according to the IIM Score. Upon completion of the experimental protocol, fresh spleens were dissected and promptly weighed to determine the spleen index.

Myositis mice are scored from 0 to 3 based on disease severity, with intermediate 0.5-point increments. A score of 0 indicates normal muscle function, posture, movement, vocalization, grip strength, and eating. Mild impairment (0.5-1) involves slightly delayed movement, weaker vocalizations, reduced grip strength, and occasional postural abnormalities or chewing difficulty. Moderate scores (1.5-2) reflect markedly reduced activity, clumsy movement, very weak or absent vocalizations, significantly weakened grip, frequent chewing difficulty, raised posture, head drooping, and tremors. Severe disease (2.5-3) presents with minimal activity, no vocalizations, complete loss of grip strength, inability to eat or support body weight, prolonged proneness, severe postural abnormalities, head drooping, and whole-body weakness, approaching a moribund state.

### Serology

Serum collected at the experimental endpoint was subjected to serological analysis. The activities of creatine kinase (CK; Solarbio, BC1145), lactate dehydrogenase (LDH; Solarbio, BC0685), and aspartate aminotransferase (AST; Solarbio, BC1565), as well as the levels of BAFF (Jonlnbio, JL20135) and anti-Jo-1 antibody (Lianbokebio, LBK-M01510) in the serum, were determined using commercial kits.

### Histopathology

Left lung and muscle tissues were fixed by immersion in 4% paraformaldehyde, followed by paraffin embedding and sectioning into 5-μm slices. The muscle sections were then stained with hematoxylin and eosin (H&E). The lung sections were stained with H&E, Masson’s trichrome, and Picrosirius red according to standard protocols. Images were acquired using a Nikon microscope, and quantitative analysis of the lung fibrotic area (from H&E staining) and collagen volume fraction (from Masson’s trichrome and Picrosirius red staining) was performed using ImageJ software.

### Micro-computed tomography scanning

On day 35, lung morphology was assessed by micro-CT using a Skyscan 1176 system (Bruker, Germany). Acquired images were subsequently reconstructed in three dimensions using the manufacturer’s proprietary software. Visual analysis involved the examination of cross-sectional, sagittal, and coronal views to evaluate the presence and morphological characteristics of pulmonary interstitial lesions.

### Lung function testing

The pulmonary function of mice was evaluated using the AniRes2005 system (Beijing BoiLab, China). After anesthesia and tracheostomy, the animals were connected to a ventilator on a body plethysmography platform, and the parameters were recorded by real-time software.

### Measurement of hydroxyproline content

The content of lung hydroxyproline, which reflects collagen deposition, was quantified via colorimetry. Specifically, dried lung tissue was hydrolyzed in 6M HCl at 120°C. Subsequently, the hydrolysate was neutralized, oxidized with chloramine-T, and then reacted with p-dimethylaminobenzaldehyde. The absorbance at 577 nm was measured, and the concentration was ascertained using a standard curve.

### Western blot analysis

After extraction from tissues, total protein concentrations were measured with a Bicinchoninic Acid (BCA) assay kit. Subsequently, the proteins were separated via Sodium Dodecyl Sulfate-Polyacrylamide Gel Electrophoresis (SDS-PAGE) and transferred onto Polyvinylidene Fluoride (PVDF) membranes. Following blocking, the membranes were incubated with primary antibodies against overnight at 4°C: Fibronectin (FN; CST, 63779S), Collagen I (COL-1; CST, 72026S), α-SMA (Affinity, AF1032), CD19 (Affinity, DF7030), CD138 (Affinity, DF6367), P-NF-κB (Affinity, AF3387), P-PI3K (Affinity, AF3241), P-AKT (Affinity, AF0016), P-STAT3 (Affinity, AF3293), P-CREB (Affinity, AF3189), NF-κB (Affinity, AF5006), PI3K (Affinity, AF6241), AKT (Affinity, AF6261), STAT3 (Affinity, AF6294), CREB (Affinity, AF6188), and GAPDH (Affinity, AF7021). After the washing procedure, the samples were then incubated for 1 hour at room temperature with HRP-conjugated secondary antibodies (Abcam, ab97051). Subsequently, protein bands were visualized via chemiluminescence and analyzed using ImageJ software.

### Quantitative real-time polymerase chain reaction

cDNA was synthesized from total RNA isolated with Trizol reagent, and qPCR was performed. The relative expression of target genes, normalized to Gapdh, was determined by the 2^-ΔΔCT method. All primer sequences used are shown in **Table 1**.

**Table 1:** Primer sequences employed in the qRT-PCR assay.

Gene	Primer	Sequence (5’-3’)
*Gapdh-Mouse*	Forward Reverse	AGGTCGGTGTGAACGGATTTG GGGGTCGTTGATGGCAACA
*Fn-Mouse*	Forward Reverse	ATGTGGACCCCTCCTGATAGT GCCCAGTGATTTCAGCAAAGG
*Col-Ⅰ;-Mouse*	Forward Reverse	GCTCCTCTTAGGGGCCACT ATTGGGGACCCTTAGGCCAT
*Acta-2-Mouse*	Forward Reverse	CCCAGACATCAGGGAGTAATGG TCTATCGGATACTTCAGCGTCA
*Tnf-α-Mouse*	Forward Reverse	CAGGCGGTGCCTATGTCTC CGATCACCCCGAAGTTCAGTAG
*Il-1β-Mouse*	Forward Reverse	GAAATGCCACCTTTTGACAGTG TGGATGCTCTCATCAGGACAG
*Il-6-Mouse*	Forward Reverse	CTGCAAGAGACTTCCATCCAG AGTGGTATAGACAGGTCTGTTGG
*CD19-Mouse*	Forward Reverse	CTTGGTATCGAGGTAACCAGTCA ACAATCACTAGCAAGATGCCC
*CD20-Mouse*	Forward Reverse	GCTCCAAAAGTGAACCTCAAAAG CCCAGGGTAATATGGAAGAGGC
*BAFF-Mouse*	Forward Reverse	CCACCGTGCCTCTGTTTTTG CTTCTGCGGAGTGATGGGAT
*Prdm1-Mouse*	Forward Reverse	GGCTCCACTACCCTTATCCTG GTTGCTTTCCGTTTGTGTGA
*Xbp-1-Mouse*	Forward Reverse	TGACGAGGTTTCAGAGGTG TGCAGAGGTGCACATAGTCTG
*Irf4-Mouse*	Forward Reverse	ACAGCACCTTATGGCTCTCTG ATGGGGTGGCATCATGTAGT

### Immunohistochemistry

Immunohistochemical detection was carried out utilizing an immunohistochemical kit (ABclonal, RK05872).

### Collection and detection of bronchoalveolar lavage fluid

BALF was collected via tracheal cannulation, centrifuged, and the supernatant stored for cytokine (ELISA) and protein (BCA) analysis. The cells were lysed and resuspended, then counted and smears were made. Cell smears were subjected to H&E staining for the purpose of differential cell counting (macrophages, lymphocytes, neutrophils).

### Retrieval of single-cell RNA sequencing database

To explore the expression pattern of PDE4B in the IIM-ILD, we utilized the single-cell RNA sequencing public database (http://www.ipfcellatlas.com/), and conducted a comparative evaluation of PDE4B expression levels across various lung tissue cell types (including alveolar epithelial cells, immune cells and pulmonary fibroblasts), thereby elucidating its cell-specific expression patterns.

### Immunofluorescence staining

Lung tissue sections underwent deparaffinization and rehydration processes, followed by antigen retrieval. After blocking, sections were incubated overnight at 4°C with primary antibodies, including combinations of PDE4B, PCNA, BAX, CD138, P-NF-κB, P-AKT, P-CREB, and P-STAT3 with CD19. Following washing, fluorescent secondary antibodies (FITC and TRITC) were applied. Nuclei were stained with DAPI, and images were captured by confocal microscopy. Immunofluorescence areas were quantified using ImageJ.

### Flow cytometric analysis

Single-cell suspensions were prepared from fresh lung tissue by mechanical disruption and enzymatic digestion. Following Fc receptor blockade, cells were stained on ice with a FITC-conjugated anti-CD19 antibody (BioLegend, Cat.152403). After washing, cells were resuspended in FACS buffer and acquired on a BD LSRFortessa flow cytometer. Data analysis was performed using FlowJo software (version 10). The gating strategy employed for the identification and analysis of lung-infiltrating B cells is as follows. First, single cells were gated based on forward scatter (FSC) area versus height to exclude cell doublets and aggregates. Second, viable cells were selected by gating on the main population in the FSC-A versus side scatter (SSC-A) plot to exclude debris and dead cells. From this population, lymphocytes were further identified based on their characteristic low SSC and intermediate FSC profile. Finally, B cells were specifically identified and quantified by gating on cells positive for the surface marker CD19 (stained with FITC-conjugated anti-CD19 antibody).

### Determination of cAMP levels

Lung tissues were finely minced and homogenized, and the obtained homogenates were employed for cAMP quantification. The levels of cAMP were ascertained using a commercial cAMP ELISA kit (Jonlnbio, JL13362).

### Statistical analysis

Data are presented as mean ± SEM with individual data points overlaid on bar graphs. A p-value less than 0.05 was deemed statistically significant. For comparisons among multiple groups, one-way ANOVA with Tukey’s *post-hoc* test for multiple comparisons was conducted using GraphPad Prism 9.5. This approach controls for Type I error inflation across the compared groups. All disease scoring, histopathological evaluation, and image quantitative analysis were performed by investigators unaware of the grouping status (using single-blind or double-blind design) to ensure the objectivity of the experimental results.

## Results

### Nerandomilast effectively alleviates idiopathic inflammatory myopathy in the model of IIM-ILD

**Figure 1:**
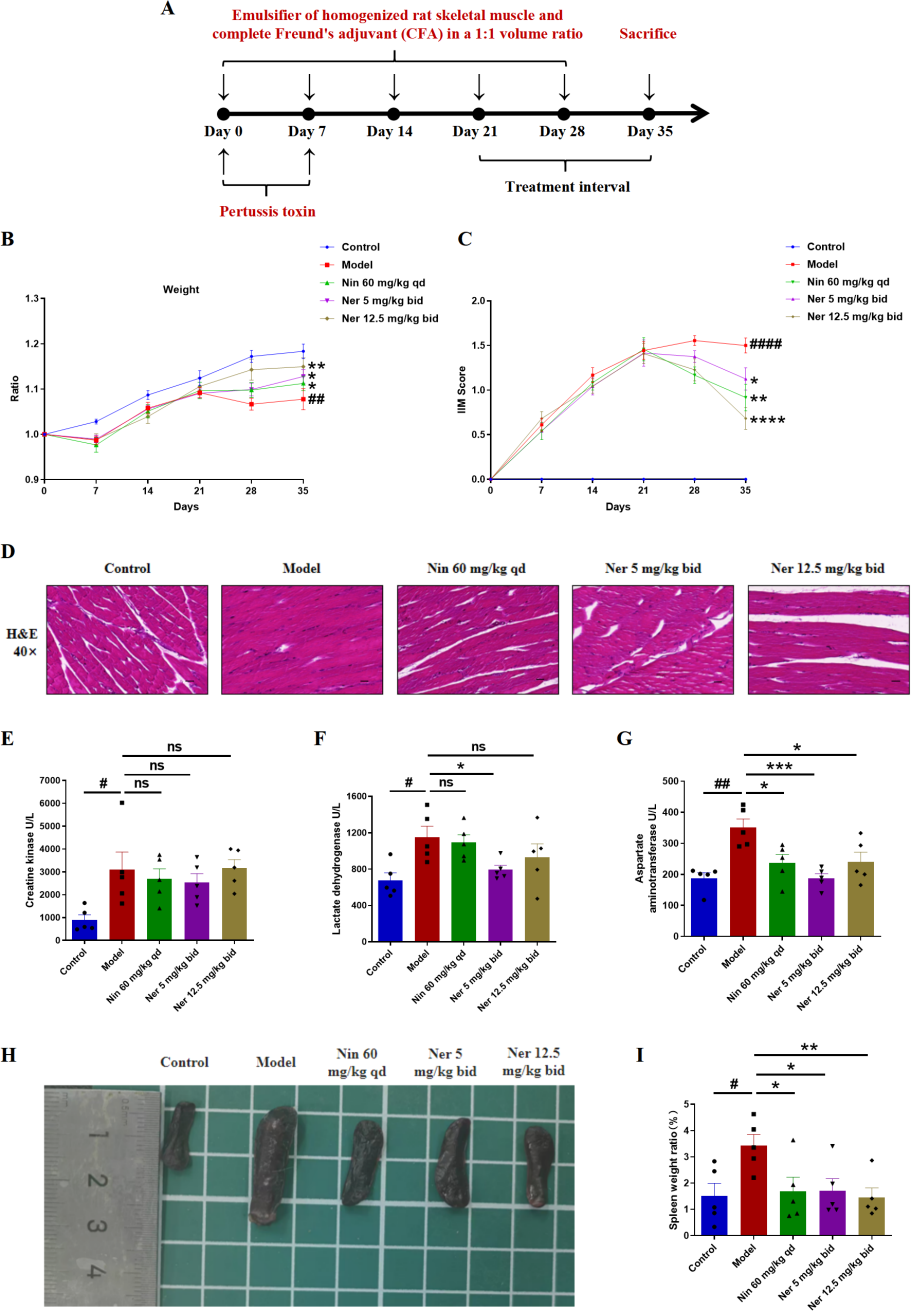
Nerandomilast effectively alleviates idiopathic inflammatory myopathy in the model of IIM-ILD. **(A)** Experimental Outline. **(B)** Body weight changes. **(C)** IIM score. **(D)** Representative muscle sections were subjected to staining with H&E (40× magnification, scale bar = 25 μm). **(E–G)** Levels of three types of serum myosin kinase. **(H)** Visual representation of the spleen’s morphology. **(I)** Spleen organ index. Data are presented as mean ± SEM (n = 5). The symbol # was used to denote statistically significant differences between the model and control groups, and asterisks (*) were employed to represent statistically significant differences between the model and treatment groups. ^#^p < 0.05, ^##^p < 0.01, ^####^p < 0.0001, *p < 0.05, **p < 0.01, ***p < 0.001, ****p < 0.0001.

To evaluate the therapeutic potential of Nerandomilast, we first assessed its effects on myositis in a stable IIM-ILD mouse model (model generation schema: **Figure 1A**). Treatment with Nerandomilast significantly ameliorated the reduction in body weight observed in the model group (**Figure 1B**). Comprehensive evaluation using the Lennon appearance assessment method further revealed that Nerandomilast mitigated key disease manifestations, including the elevated myositis scores, reduced activity levels, extensive dorsal ulceration, and decreased vocalization seen in the model group (**Figure 1C**). Histopathological examination confirmed substantial inflammatory cell infiltration in the muscle tissue of model mice, a phenotype that was effectively suppressed by Nerandomilast (**Figure 1D**). Furthermore, we measured serum biomarkers of muscle injury-creatine kinase (CK), lactate dehydrogenase (LDH), and aspartate aminotransferase (AST)-to assess the severity of myositis. The results revealed elevated levels of all three biomarkers in the IIM-ILD model group, indicating active muscle inflammation and injury in IIM-ILD. Treatment with Nerandomilast significantly reduced serum AST levels and showed a certain trend of reduction in CK and LDH (though without significant differences), suggesting its ameliorating effect on muscle injury (**Figures 1E–G**). Notably, further studies revealed that mice in the IIM-ILD model exhibited splenomegaly (increased spleen-to-body weight ratio), which may result from extensive lymphocyte proliferation and aggregation, suggesting that IIM-ILD manifests systemic immune abnormalities rather than localized muscle lesions. Nerandomilast significantly ameliorated splenomegaly, implying its potential role as a systemic immunomodulator that regulates systemic lymphocyte homeostasis (**Figures 1H, I**). In summary, these results demonstrate that Nerandomilast effectively ameliorates the myositis phenotype in the IIM-ILD model and may potentially improve systemic inflammation.

### Nerandomilast successfully mitigates pulmonary fibrosis in IIM-ILD mice

**Figure 2:**
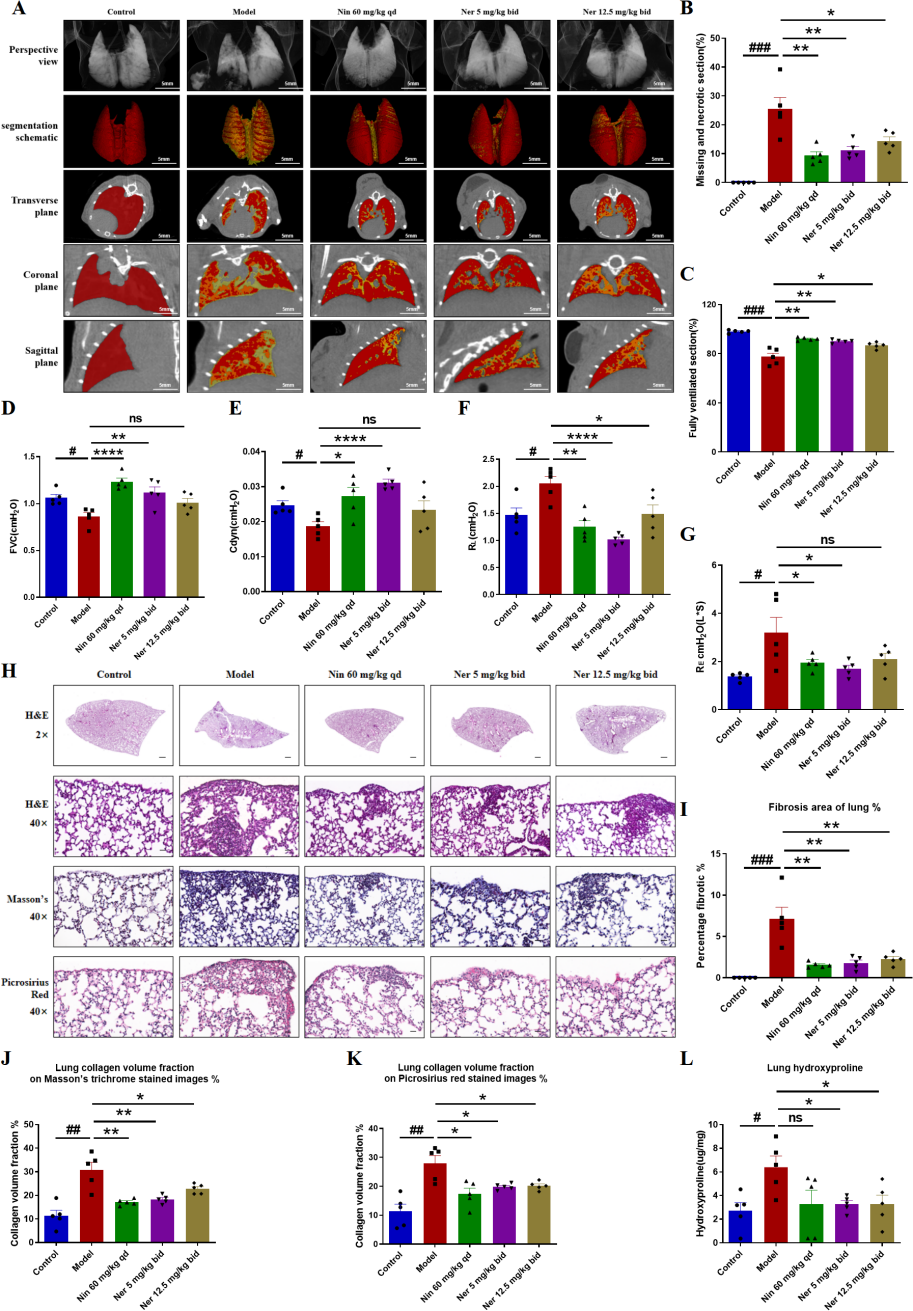
Nerandomilast successfully mitigates pulmonary fibrosis in IIM-ILD mice. **(A–C)** Lung CT images, segmentation schematic, and quantitative analysis. **(D–G)** Pulmonary Function Parameters. **(H)** Representative lung sections were subjected to staining with H&E (2× magnification, scale bar = 500 μm; 40× magnification, scale bar = 25 μm), Masson’s trichrome, and Picrosirius red. **(I)** The proportion of lung fibrosis region according to the H&E staining results in **(H)**. **(J, K)** Measurement of the collagen density in lung tissue based on the results shown in **(H)**. **(L)** Hydroxyproline Content. Data are presented as mean ± SEM (n = 5). ^#^p < 0.05, ^##^p < 0.01, ^###^p < 0.001, *p < 0.05, **p < 0.01, ****p < 0.0001.

**Figure 3:**
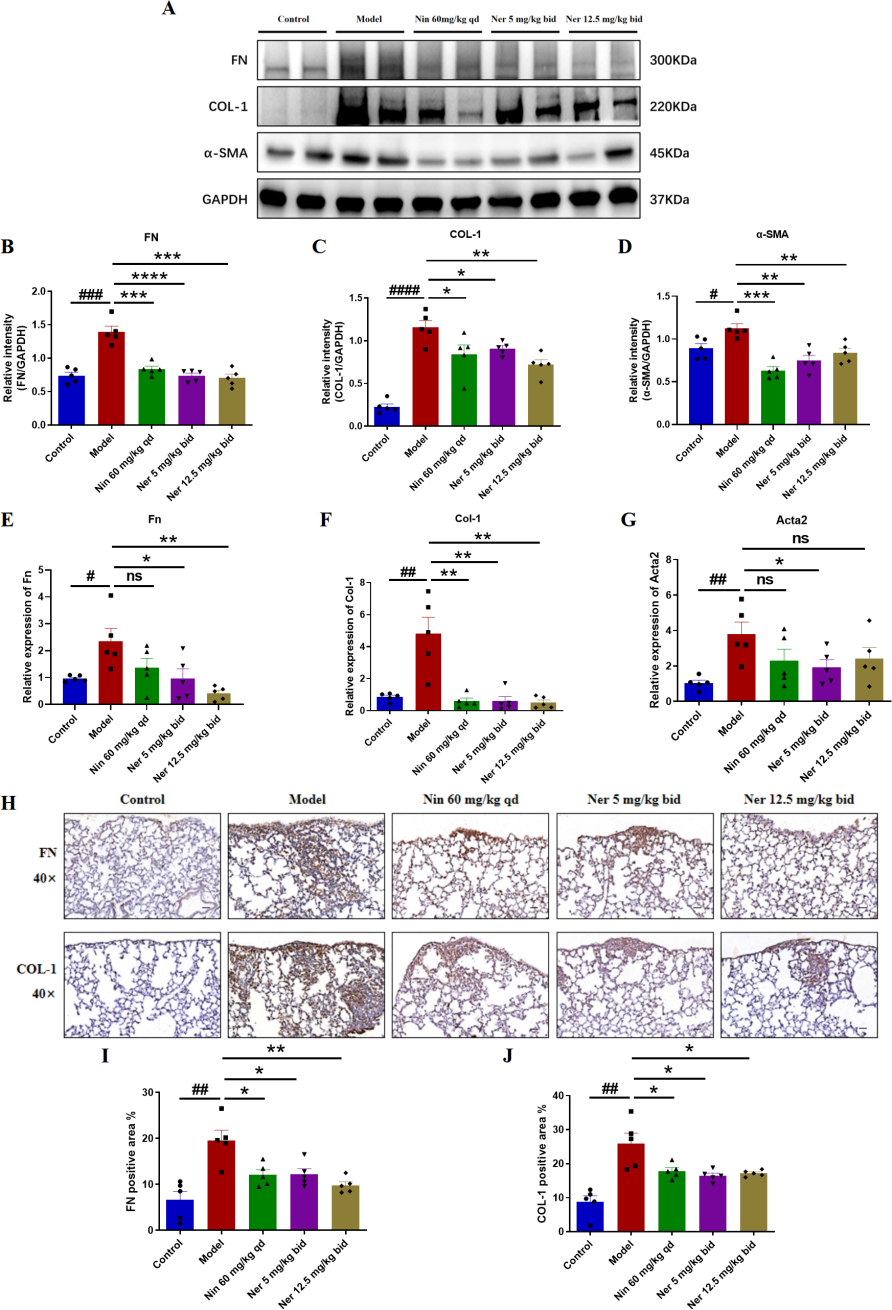
Nerandomilast significantly inhibits the expression of pulmonary fibrosis markers in IIM-ILD mice. **(A–D)** Western blot analysis of FN, COL-1, and α-SMA protein expression levels in lung tissue. **(E–G)** qRT-PCR was used to detect the mRNA expression levels of *Fn*, *Col-1*, and *α-SMA* (encoded by the Acta2 gene) in lung tissue. **(H–J)** Immunohistochemical staining of FN, and COL-1 in lung tissue (40× magnification, scale bar = 25 μm). Data are presented as mean ± SEM (n = 5). ^#^p < 0.05, ^##^p < 0.01, ^###^p < 0.001, ^####^p < 0.0001, *p < 0.05, **p < 0.01, ***p < 0.001, ****p < 0.0001.

We further focused on the effect of Nerandomilast on pulmonary fibrosis in IIM-ILD mice. Micro-CT and pulmonary function tests can more directly assess the extent of pulmonary structural damage and functional impairment. CT imaging demonstrated that Nerandomilast significantly increased lung ventilation while reducing necrotic areas and tissue defects (**Figures 2A–C**). Pulmonary function evaluation revealed that low-dose Nerandomilast markedly improved forced vital capacity (FVC) and dynamic compliance (Cdyn), while simultaneously decreasing inspiratory airway resistance (RL) and expiratory airway resistance (RE) (**Figures 2D–G**). Notably, high-dose Nerandomilast showed improvement in pulmonary function parameters but did not reach statistical significance, suggesting that in this IIM-ILD model, the low dose may have reached a pharmacodynamic plateau for maximal pulmonary function improvement, and the high-dose group exhibited greater data variability, which may also affect statistical significance. Pathological staining of lung tissue and hydroxyproline content determination are the most classic and complementary “gold standard” methods for evaluating pulmonary fibrosis. H&E staining results confirmed that Nerandomilast significantly reduced the area of fibrotic regions in the lung tissue of IIM-ILD model mice and improved the inflammatory cell infiltration and destructive thickening of the alveolar septa. Masson’s trichrome and Picrosirius red staining further revealed that the therapeutic effects of Nerandomilast were accompanied by a significant reduction in pathological collagen deposition in the lung interstitium, particularly mature type I collagen, which is a hallmark of advanced fibrosis (**Figures 2H–K**). Further quantitative analysis of hydroxyproline content (a direct indicator of collagen overproduction) directly demonstrated that Nerandomilast treatment significantly reduced the total collagen level in the lungs, providing corroborative evidence for the aforementioned morphological observations (**Figure 2L**). Subsequently, we detected the expression of fibrosis markers-FN (Fibronectin), COL-1 (Collagen I), and α-SMA (α-Smooth Muscle Actin)-at the molecular level in lung tissue. WB (**Figures 3A–D**) and qRT-PCR (**Figures 3E–G**) results, respectively, confirmed from protein and mRNA perspectives that Nerandomilast could inhibit the expression of these three fibrosis markers. Immunohistochemical analysis further visually revealed that Nerandomilast reduced the deposition of FN and COL-1 and the number of α-SMA-positive cells in the fibrotic lesion areas (**Figures 3H–J**). In conclusion, these results demonstrate that Nerandomilast effectively alleviates pulmonary fibrosis in IIM-ILD mice.

### Nerandomilast effectively improves pulmonary inflammation in the model of IIM-ILD

**Figure 4:**
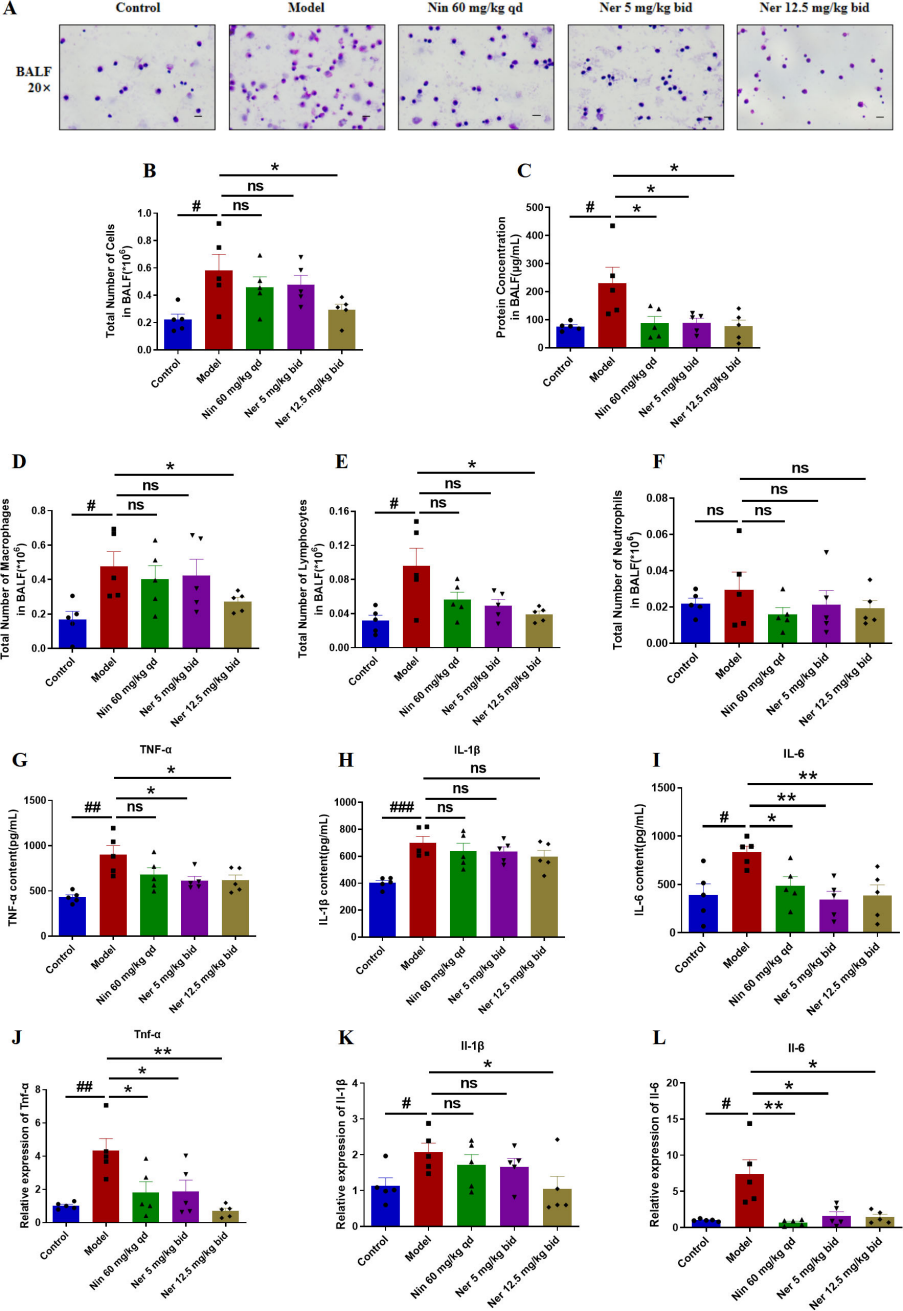
Nerandomilast effectively improves pulmonary inflammation in the model of IIM-ILD. **(A)** Microscopic observation of BALF smears (20× magnification; scale bar = 50 μm). **(B)** Total cell count in BALF. **(C)** Protein concentration in BALF. **(D–F)** Differential cell counts of macrophages, lymphocytes, and neutrophils in BALF. **(G–I)** Levels of TNF-α, IL-1β, and IL-6 in BALF detected by ELISA. **(J–L)** qRT-PCR was used to detect the mRNA expression levels of *Tnf-α*, *Il-1β*, and *Il-6* in lung tissue. Data are presented as mean ± SEM (n = 5). ^#^p < 0.05, ^##^p < 0.01, ^###^p < 0.001, *p < 0.05, **p < 0.01.

Considering the critical role of inflammation in driving fibrotic progression, we also investigated whether Nerandomilast could alleviate pulmonary inflammation in IIM-ILD mice. The assessment of inflammatory cells and mediators in the local immune microenvironment of the lungs was conducted through BALF cell counts and analysis of inflammatory cytokines (TNF-α, IL-1β, IL-6, etc.), thereby exploring the effects of Nerandomilast on pulmonary immune cell infiltration and inflammatory responses. BALF analysis revealed that Nerandomilast reduced the elevation of disease-induced total cell counts (**Figures 4A, B**) and total protein concentrations (**Figure 4C**). Additionally, Nerandomilast decreased the number of lymphocytes in BALF, had a mild effect on macrophages, and showed almost no impact on neutrophils, suggesting a regulatory effect of Nerandomilast on lymphocytes (**Figures 4D–F**). At the molecular level, Nerandomilast lowered the levels of TNF-α and IL-6 in BALF (**Figures 4G–I**) and inhibited their mRNA expression in lung tissue (**Figures 4J–L**). Notably, Nerandomilast did not significantly suppress IL-1β levels, implying that the IL-1β/NLRP3 pathway may not be the primary therapeutic mechanism of Nerandomilast in the IIM-ILD model. In conclusion, these results demonstrate that Nerandomilast can ameliorate pulmonary inflammation in the IIM-ILD model.

### Nerandomilast can modulate B cells in the lung tissue of IIM-ILD model mice

**Figure 5:**
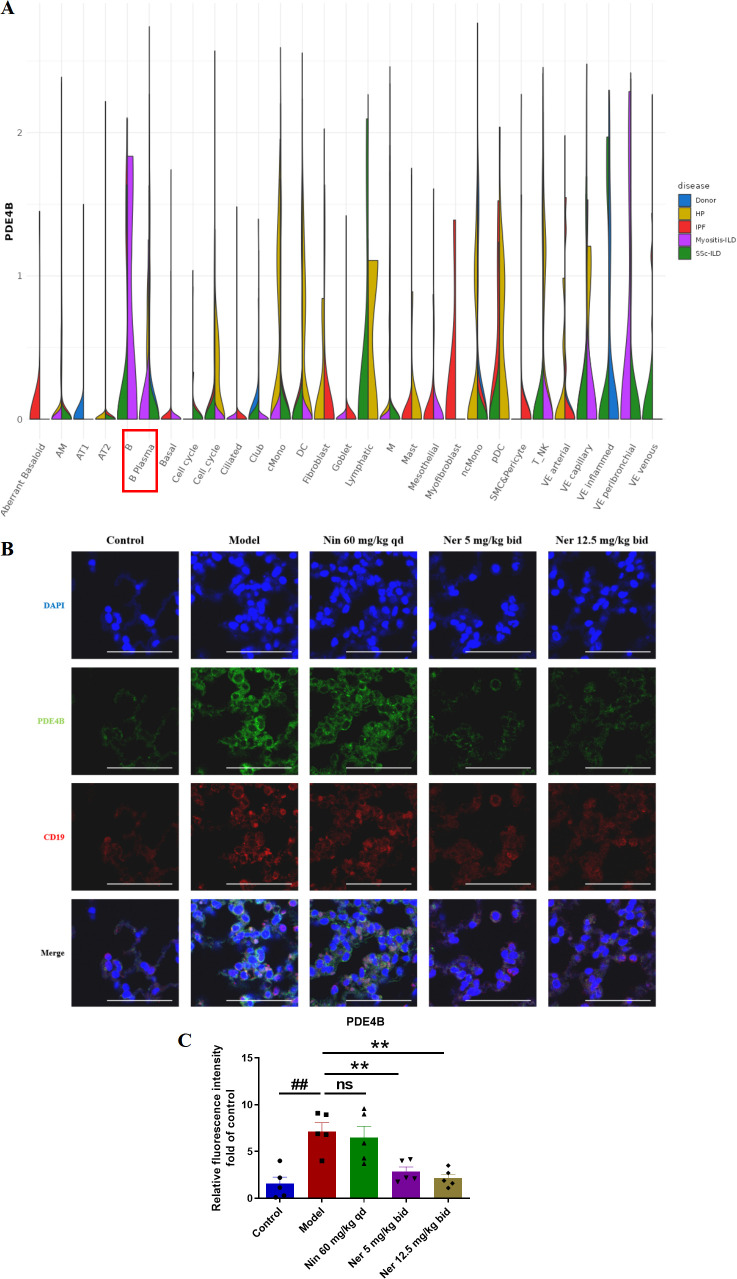
Nerandomilast significantly inhibits the expression of PDE4B in the lung tissue of IIM-ILD mice. **(A)** The expression of PDE4B in different cells of Myositis-ILD by IPF Cell Atlas Database. **(B, C)** Immunofluorescence staining of PDE4B and CD19 in lung tissue (scale bar = 50 μm). Data are presented as mean ± SEM (n = 5). ^##^p < 0.01, **p < 0.01.

Next, we aim to elucidate the mechanism by which Nerandomilast improves IIM-ILD. Through mining and analyzing the IPF cell atlas database, we found that in the context of myositis-ILD, the expression level of PDE4B in B cells was significantly higher than in other lung tissue cell types (**Figure 5A**), suggesting that B cells may be a key target of the PDE4B signaling pathway in myositis. To validate this finding in an *in vivo* model, we performed immunofluorescence co-localization experiments. The results showed that compared to the control group, the expression of PDE4B protein in CD19^+^ B cells in the lung tissue of IIM-ILD model mice was significantly upregulated; however, this abnormally high expression was markedly suppressed after Nerandomilast treatment, suggesting that Nerandomilast can target and regulate PDE4B molecules in B cells within the pathological lung tissue (**Figures 5B, C**). Notably, the significant alleviation of splenomegaly observed in our previous phenotypic data, as well as the reduction in lymphocyte counts in BALF, further corroborate the potential immunomodulatory effects of Nerandomilast on lymphocytes at both systemic immune and local pulmonary microenvironment levels. Based on the aforementioned database clues, protein expression validation, and phenotypic associations, we subsequently focused our research on systematically investigating the regulatory effects of Nerandomilast on B cell function and its downstream mechanisms.

### Nerandomilast significantly inhibits B cells proliferation and activation in lung tissue in the model of IIM-ILD

**Figure 6:**
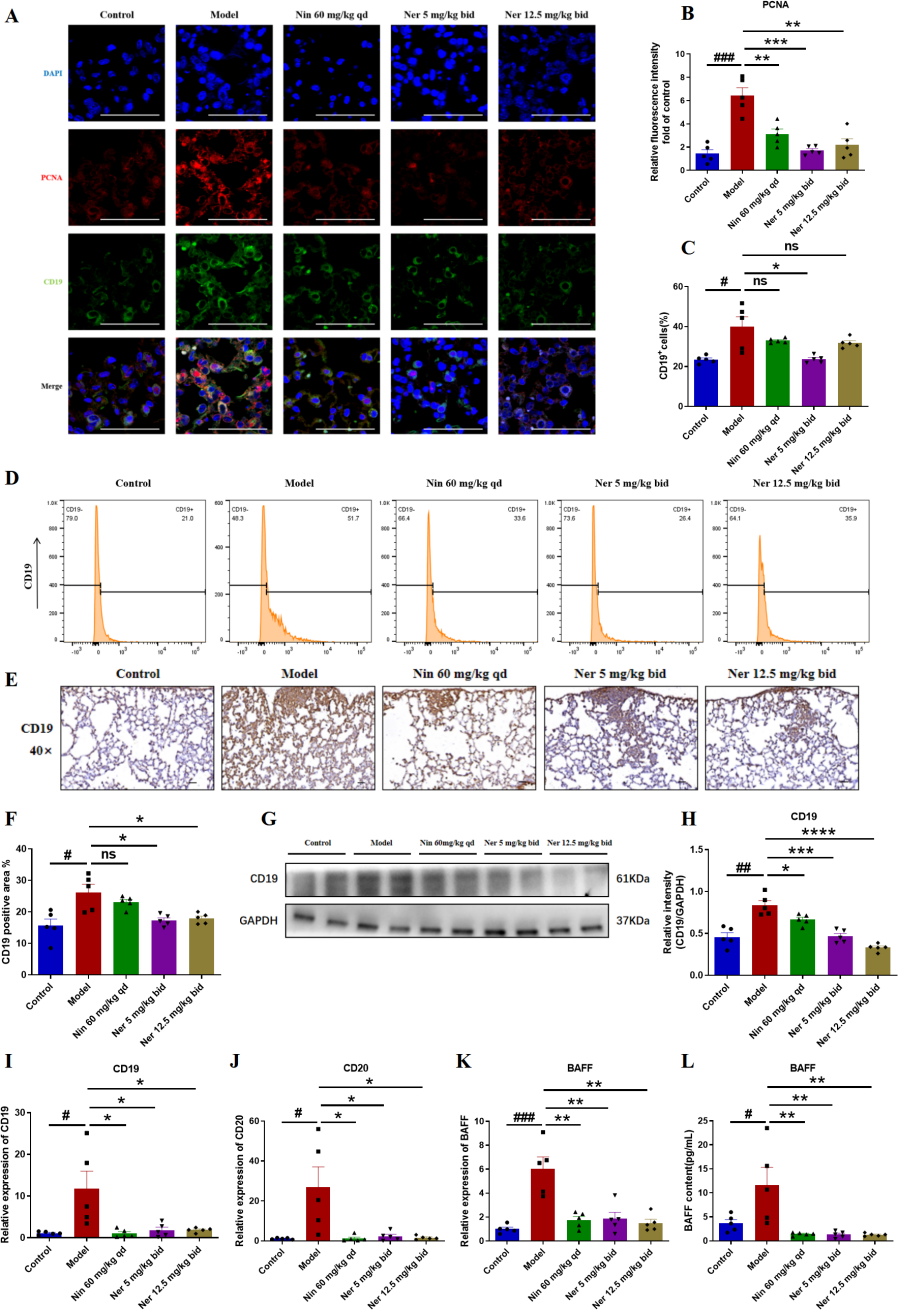
Nerandomilast significantly inhibits B cells proliferation and activation in lung tissue in the model of IIM-ILD. **(A, B)** Immunofluorescence staining of PCNA and CD19 in lung tissue (scale bar = 50 μm). **(C, D)** Flow cytometry analysis. **(E, F)** Immunohistochemical staining of CD19 in lung tissue (40× magnification, scale bar = 25 μm). **(G, H)** Western blot analysis of CD19 protein expression levels in lung tissue. **(I–K)** qRT-PCR was used to detect the mRNA expression levels of *CD19*, *CD20*, and *BAFF* in lung tissue. **(L)** Levels of BAFF in the serum. Data are presented as mean ± SEM (n = 5). ^#^p < 0.05, ^##^p < 0.01, ^###^p < 0.001, *p < 0.05, **p < 0.01, ***p < 0.001, ****p < 0.0001.

To define Nerandomilast’s regulatory influence on B cells, we first examined its effects on their proliferation and activation. Co-localization immunofluorescence analysis of PCNA (proliferating cell nuclear antigen) and CD19 revealed a significant population of CD19^+^PCNA^+^ double-positive B cells in the lung tissues of IIM-ILD model mice, confirming the presence of active B cell dysproliferation under disease conditions. Treatment with Nerandomilast significantly reduced the proportion of CD19^+^PCNA^+^ double-positive cells (**Figures 6A, B**). We simultaneously performed co-localization analysis of BAX (apoptosis-inducing protein) and CD19 to investigate whether the drug also induces apoptosis to eliminate B cells. Compared to the model group, the proportion of CD19^+^BAX^+^ double-positive cells did not show statistically significant elevation in the Nerandomilast treatment group (**Supplementary Figure S1A**). This result indicates that under the current study model and time point, Nerandomilast did not significantly activate the BAX-mediated apoptosis pathway in B cells. In the lungs of IIM-ILD model mice, flow cytometry identified a significant elevation in the percentage of CD19^+^ B cells, which showed a decreasing trend upon Nerandomilast treatment, although the high-dose Nerandomilast group did not reach statistical significance (**Figures 6C, D**). Immunohistochemistry, however, confirmed extensive CD19^+^ B cell infiltration, a pathology that was substantially alleviated by Nerandomilast (**Figures 6E, F**). The suppressive effect of Nerandomilast on B cells was consistently observed at the molecular level, as it reversed the upregulation of CD19 protein expression (**Figures 6G-H**) and CD19/CD20 mRNA expression (**Figures 6I, J**). The apparent discrepancy between flow cytometry (cell percentage) and Western blot (total protein) may reflect not only changes in B cell numbers but also a potential downregulation of CD19 protein expression per cell, indicative of altered B cell status. BAFF (B cell activation factor) is a key factor for B cell activation, survival, and class switching. Interestingly, we found that the mRNA expression of BAFF in the lung tissue of IIM-ILD model mice was significantly higher than that in the control group, indicating that B cells were in an activated state. After treatment with Nerandomilast, this hyperexpression was effectively reversed (**Figure 6K**). Consistent with this, the serum BAFF concentration in model mice was also significantly upregulated, while Nerandomilast significantly reduced serum BAFF levels (**Figure 6L**), suggesting that Nerandomilast can systematically reduce systemic BAFF levels, decrease pulmonary B cell infiltration, and inhibit abnormal B cell activation. Collectively, these findings indicate that Nerandomilast exerts an inhibitory effect on B cell accumulation and activation in the IIM-ILD model.

### Nerandomilast exerts a notable inhibitory effect on the differentiation of B cells into plasma cells and the production of autoantibodies in the lung tissue within IIM-ILD

**Figure 7:**
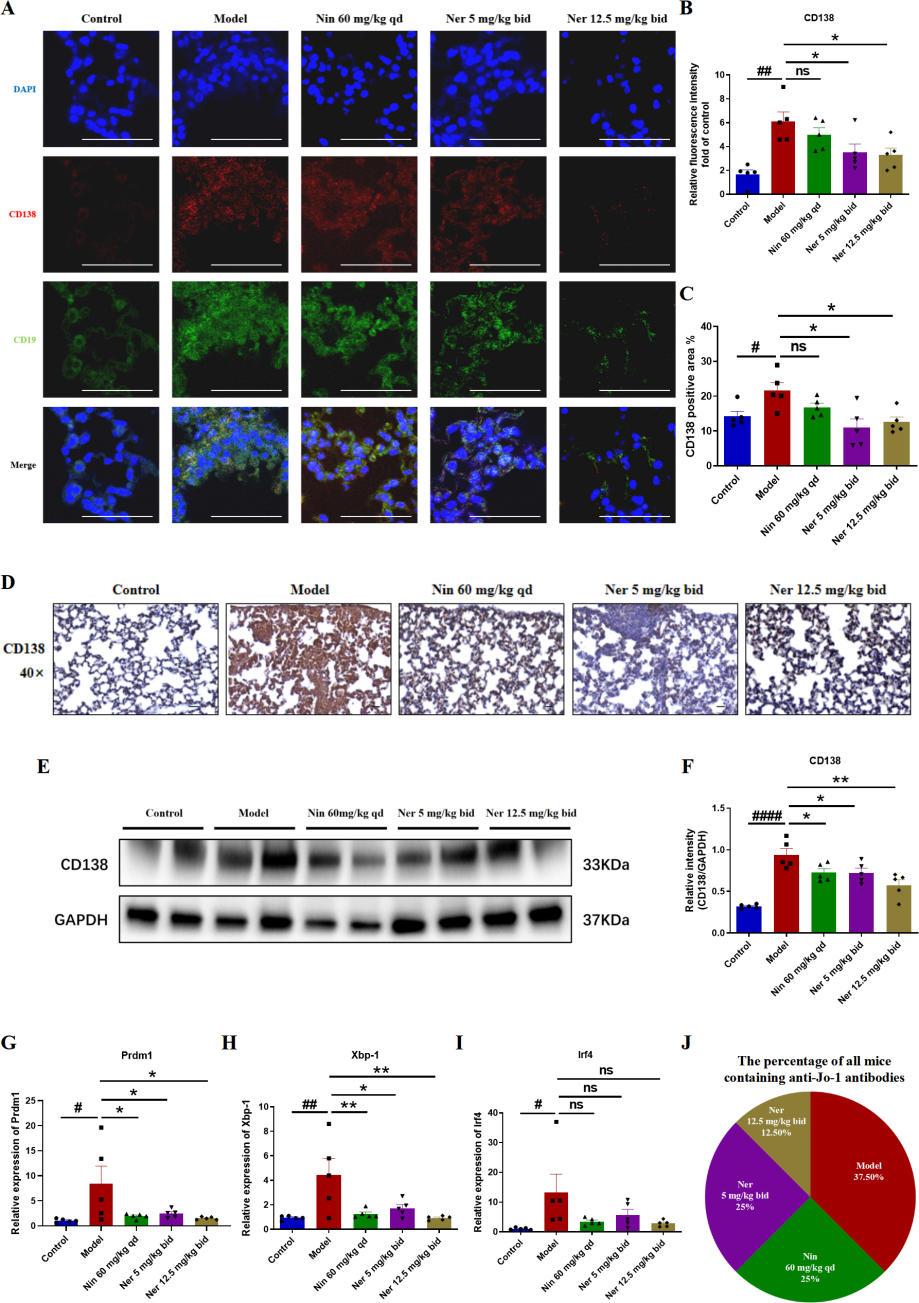
Nerandomilast exerts an inhibitory effect on the differentiation of B cells into plasma cells and the production of autoantibodies in the lung tissue within IIM-ILD. **(A, B)** Immunofluorescence staining of CD138 and CD19 in lung tissue (scale bar = 50 μm). **(C, D)** Immunohistochemical staining of CD138 in lung tissue (40× magnification, scale bar = 25 μm). **(E, F)** Western blot analysis of CD138 protein expression levels in lung tissue. **(G–I)** qRT-PCR was used to detect the mRNA expression levels of *Prdm1*, *Xbp-1*, and *Irf4* in lung tissue. **(J)** The percentage of all mice containing anti-Jo-1 antibodies in serum detected by ELISA. Data are presented as mean ± SEM (n = 5). ^#^p < 0.05, ^##^p < 0.01, ^####^p < 0.0001, *p < 0.05, **p < 0.01.

Subsequently, we evaluated the ability of Nerandomilast to inhibit B cell differentiation into antibody-secreting plasma cells and subsequent pathogenic autoantibody production. Immunofluorescence and immunohistochemical staining demonstrated a significant increase in CD138^+^ plasma cell infiltration in the lung tissues of model mice, suggesting that in IIM-ILD, B cells are not only activated and proliferated but also abnormally and extensively differentiated into terminal plasma cells. Nerandomilast significantly alleviated this phenomenon, indicating its regulatory effect on plasma cells (**Figures 7A–D**). Consistent with these observations, Western blot analysis confirmed that Nerandomilast treatment downregulated the expression of the plasma cell-specific marker CD138 in lung tissue homogenates (**Figures 7E, F**). We then assessed the expression of key transcription factors regulating plasma cell differentiation. qRT-PCR results revealed significant dysregulation of critical regulatory genes for plasma cell differentiation in the lung tissues of IIM-ILD model mice: the mRNA levels of Prdm1 (the primary regulator of plasma cell differentiation), Xbp-1 (an essential factor for plasma cell function), and Irf4 (an early driver of plasma cell differentiation) were all significantly upregulated. Nerandomilast significantly suppressed the abnormal expression of Prdm1 and Xbp-1 (**Figures 7G–I**), suggesting that Nerandomilast not only reduces the infiltration of CD138^+^ plasma cells at the cellular level but may also interfere with their differentiation program at the transcriptional level by synergistically inhibiting the expression of key transcription factors involved in plasma cell differentiation, thereby blocking the terminal differentiation pathway of B cells into antibody-secreting plasma cells. Interestingly, we found that Nerandomilast reduced the expression of Irf4 but did not achieve statistical significance, which may be because Irf4, as a marker of early activation, may exhibit greater fluctuations or peak expression at earlier time points. However, the detection at the end of the treatment in this experiment may have missed the most significant window for its changes.

Plasma cells are the sole source of autoantibodies, and their reduction may impair antibody production. Furthermore, we evaluated the functional impact of Nerandomilast on plasma cell suppression by measuring the serological positivity rate of myositis-associated JO-1 autoantibodies. ELISA quantification revealed that serum JO-1 autoantibody positivity, which was highest in the model group (37.5% of total positive cases), was significantly reduced in mice receiving Nerandomilast (**Figure 7J**). This suggests that Nerandomilast effectively reduces the levels of pathogenic autoantibodies produced by these plasma cells while inhibiting their local differentiation in the lungs.

In conclusion, these results indicate that Nerandomilast potently inhibits the differentiation of B cells into plasma cells and curtails autoantibody production in the IIM-ILD model.

### Nerandomilast inhibits NF-κB, PI3K-AKT, and STAT3 signaling pathways and enhances CREB signaling pathway in B cells of IIM-ILD model lung tissue

**Figure 8:**
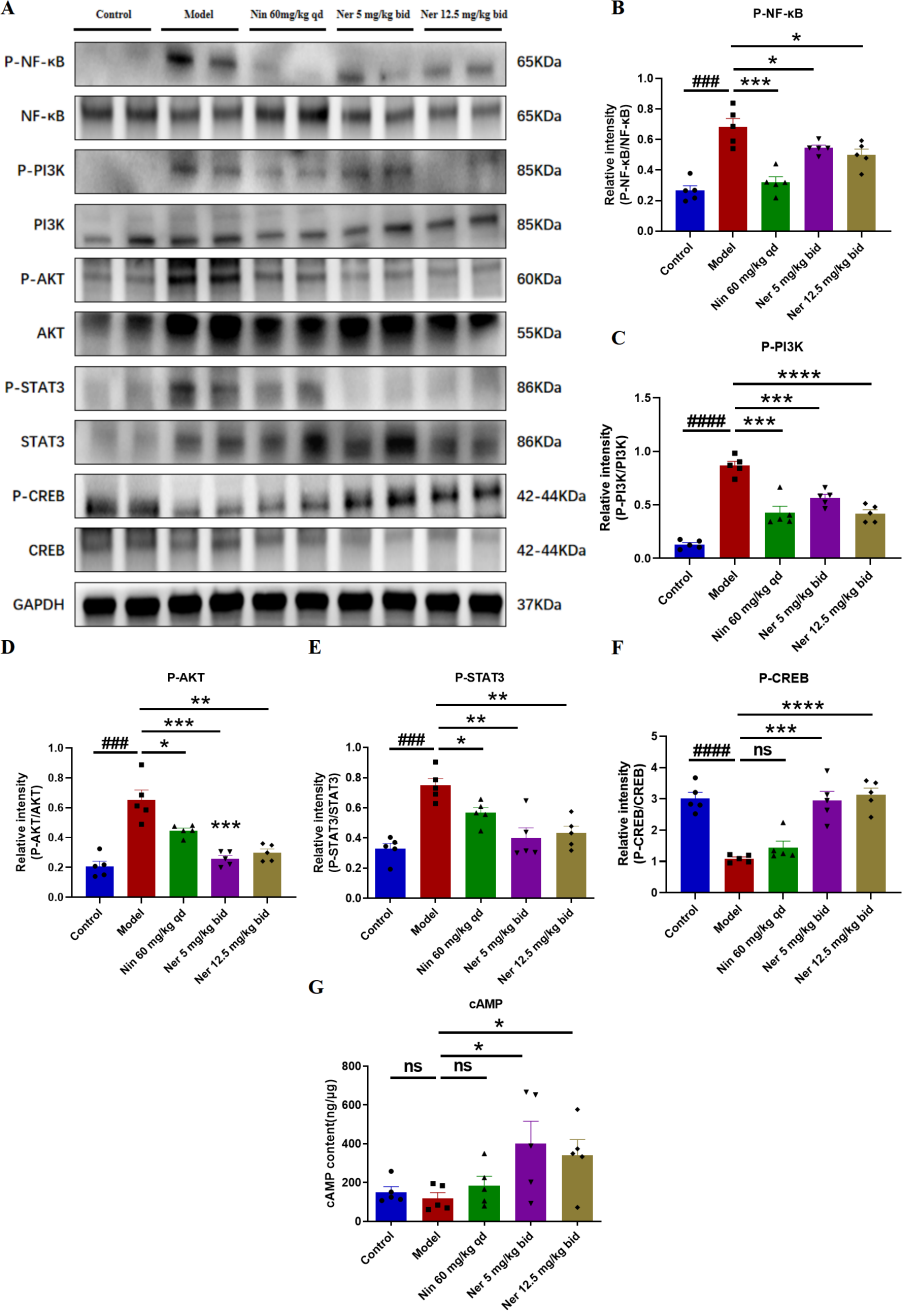
Nerandomilast inhibits NF-κB, PI3K-AKT, and STAT3 signaling pathways and enhances CREB signaling pathway in lung tissue in the model of IIM-ILD. **(A–F)** Western blot analysis of P-NF-κB, NF-κB, P-PI3K, PI3K, P-AKT, AKT, P-STAT3, STAT3, P-CREB, and CREB protein expression levels in lung tissue. **(G)** Levels of cAMP in lung tissue. Data are presented as mean ± SEM (n = 5). ^###^p < 0.001, ^####^p < 0.0001, *p < 0.05, **p < 0.01, ***p < 0.001, ****p < 0.0001.

**Figure 9:**
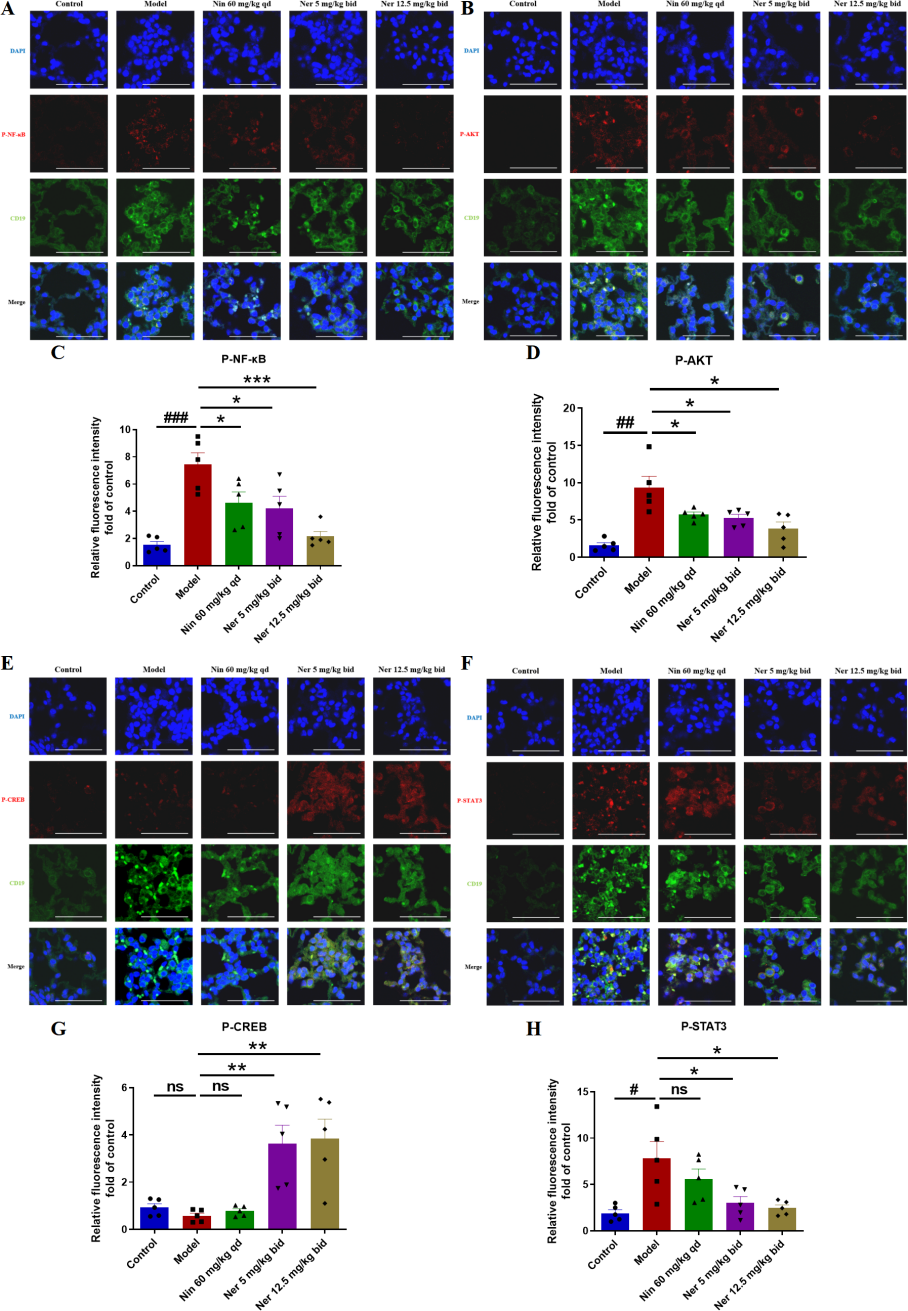
Nerandomilast inhibits NF-κB, PI3K-AKT, and STAT3 signaling pathways and enhances CREB signaling pathway in B cells of IIM-ILD model lung tissue. **(A–H)** Co-localization immunofluorescence staining of P-NF-κB, P-AKT, P-CREB, and P-STAT3 with CD19 (scale bar = 50 μm). Data are presented as mean ± SEM (n = 5). ^#^p < 0.05, ^##^p < 0.01, ^###^p < 0.001, *p < 0.05, **p < 0.01, ***p < 0.001.

To explore the signaling basis for the phenotypic changes described above, we analyzed key pathways related to B cell activation, survival, and differentiation. Western blot results showed that Nerandomilast treatment concomitantly suppressed the phosphorylation activation of the PI3K/AKT, NF-κB, and STAT3 pathways while enhancing CREB phosphorylation (**Figures 8A–F**). The drug also significantly elevated cAMP levels in lung tissue (**Figure 8G**), consistent with the effect of PDE4B inhibition. Using immunofluorescence co-localization, we confirmed that these phosphorylation changes occurred within CD19^+^ B cells (**Figures 9A–H**). These data suggest that by elevating cAMP levels, Nerandomilast may coordinately inhibit multiple pro-inflammatory/pro-survival signaling axes and activate the CREB pathway, a negative regulator, thereby promoting the restoration of immune homeostasis.

## Discussion

IIM-ILD is a severe autoimmune condition with high mortality, for which treatment options remain limited. Phosphodiesterase-4 (PDE4), the key enzyme responsible for intracellular cAMP degradation, has the PDE4B subtype highly expressed in lung tissue and is considered a promising therapeutic target for pulmonary fibrosis (21). This study provides the first systematic evaluation of the efficacy of the highly selective PDE4B inhibitor Nerandomilast in an IIM-ILD animal model and links its therapeutic effects to the direct modulation of B cells. The principal findings can be summarized as follows: 1) Nerandomilast effectively alleviates the overall disease burden in the IIM-ILD model, including muscle inflammation, pulmonary fibrosis, and pulmonary inflammation. 2) Its efficacy is closely associated with inhibition of B cell proliferation, activation, and differentiation into plasma cells. 3) Mechanistically, by elevating cAMP levels, the drug appears to reprogram the intracellular signaling network in B cells, coordinately inhibiting the PI3K/AKT, NF-κB, and STAT3 pathways while activating the CREB pathway.

Our study initially confirmed the significant efficacy of Nerandomilast at the whole-animal level. Nerandomilast significantly reversed the weight loss, elevated myositis scores, and muscle inflammatory cell infiltration observed in mice with IIM-ILD. Notably, Nerandomilast demonstrated a more pronounced inhibitory effect on serum AST levels compared to CK and LDH. CK and LDH are relatively specific and sensitive markers of skeletal muscle injury, with their elevation directly reflecting active muscle inflammation in the model. In contrast, the elevation of AST may reflect systemic immune dysregulation, which aligns with the characteristics of IIM-ILD as a systemic autoimmune disease. We believe the more significant inhibition of AST by Nerandomilast suggests its potential to influence systemic inflammation earlier or more effectively. Additionally, Nerandomilast significantly improved splenomegaly symptoms, implying its systemic immunomodulatory effects. We hypothesize that the therapeutic effects of Nerandomilast on IIM may stem from its systemic circulation-mediated action on immune cells within the muscle (including potentially infiltrated B cells) or its indirect promotion of muscle function by improving systemic inflammatory status. Furthermore, the normalization of spleen size, as the primary site of B cell activation and proliferation, is consistent with the reduced B cell infiltration and suppressed proliferation observed in lung tissue. This suggests that Nerandomilast may regulate B cell pool homeostasis at the systemic immune level. Meanwhile, Nerandomilast demonstrated potent anti-fibrotic and anti-inflammatory effects in the lungs, manifested as reduced lung tissue loss, decreased pulmonary fibrosis area and collagen deposition, lower levels of fibrosis markers, and reduced total inflammatory cell count in BALF. In summary, these results indicate the potential therapeutic role of Nerandomilast in IIM-ILD.

Through bioinformatics analysis, we found that PDE4B was significantly overexpressed in B cells but only weakly expressed in other cell types. Further immunofluorescence staining confirmed that Nerandomilast treatment inhibited PDE4B expression in B cells of the lungs in IIM-ILD model mice. This prompted us to shift our mechanistic research focus to B cells. From a pathophysiological perspective, B cells serve as effector cells (antibodies), regulatory cells (cytokines), and bridging cells (antigen presentation), making them an ideal model for studying the association between immune dysregulation and end-organ damage. Subsequent experiments demonstrated that Nerandomilast inhibited the accumulation and activation of B cells in the lungs of IIM-ILD model mice, suggesting its potent regulatory effect on B cells. Immunofluorescence staining of B cells for proliferation and apoptosis revealed that Nerandomilast primarily reduced lung B cell populations by inhibiting B cell proliferation rather than inducing apoptosis, a finding further confirmed by immunohistochemistry and Western blotting. Notably, we observed that Nerandomilast decreased the level of BAFF, a factor often elevated in IIM-ILD patients and a key regulator of B cell activation, survival, and class switching (22), indicating potential interference with the aberrant B cell survival cycle. Of course, this does not negate the critical role of other immune cells (e.g., macrophages, T cells) and fibroblasts. In fact, Nerandomilast may exert synergistic effects through multi-target mechanisms. For instance, single-cell sequencing revealed weak PDE4B expression in macrophages of myositis-associated ILD patients, while BALF counts confirmed that Nerandomilast reduced the number of macrophages in BALF of IIM-ILD model mice. However, the core value of this study lies in elucidating the mechanism of PDE4B-mediated B-cell reprogramming—a previously overlooked mechanism—that provides new insights into drug action profiles and the development of more precise IIM-ILD treatment strategies.

To further clarify the influence of Nerandomilast on the terminal differentiation and function of B cells, our study concentrated on plasma cells (CD138^+^), which are the principal producers of autoantibodies. The differentiation of these cells is precisely regulated by key transcription factors, including Prdm1, Xbp-1, and Irf4 (23). Our investigation revealed that Nerandomilast reduced lung plasma cell infiltration, suppressed the expression of key transcription factors (Prdm1, Xbp-1, Irf4), and decreased anti-Jo-1 autoantibody positivity. This strongly suggests that Nerandomilast can curtail pathogenic autoantibody production at its source, which is crucial for controlling the autoimmune component of IIM-ILD.

The observed cellular phenotypic changes are supported by alterations in signaling. The increase in cAMP, a second messenger, is a direct consequence of PDE4B inhibition. The activation of CREB, a downstream effector of cAMP-PKA observed in this study, aligns with the known anti-inflammatory role of cAMP. More significantly, we observed the coordinated inhibition of the PI3K/AKT, NF-κB, and STAT3 pathways, which are closely related to B cell activation, survival, and differentiation. Notably, the IL-6/STAT3 axis is both a key driver of plasma cell differentiation and subject to negative regulation by the cAMP-PKA pathway (24, 25). Therefore, the inhibition of STAT3 by Nerandomilast may constitute a central node linking its anti-inflammatory effects with the inhibition of plasma cell differentiation. We propose that Nerandomilast, via the “master switch” of cAMP, may induce a systemic reset of the signaling network within B cells, shifting them from a pro-inflammatory state towards homeostasis. However, while our data indicate an association between pharmacotherapy and signaling changes, causal validation of these pathways requires further intervention studies.

A major strength of this study is the pioneering extension of the therapeutic potential of a selective PDE4B inhibitor to IIM-ILD, with a novel focus on B cells, supported by a comprehensive evidence chain spanning from overall phenotype to cellular function and molecular signaling. Nevertheless, certain limitations should be acknowledged. Firstly, regarding the representativeness of the animal model. The model used in this study effectively simulates the phenotype of anti-synthetase syndrome associated with anti-Jo-1 antibodies. However, human IIM-ILD is highly heterogeneous, encompassing various myositis-specific autoantibodies (e.g., anti-MDA5) and clinical subtypes. Therefore, our findings primarily pertain to the anti-Jo-1 positive subtype, and the efficacy of Nerandomilast in other MSA-positive subtypes requires further validation. Furthermore, inherent differences between animal models and humans in immune cell composition, migration patterns, and pulmonary tissue architecture may influence drug distribution, target accessibility, and ultimately therapeutic efficacy. Additionally, the acute/subacute progression of the animal model differs from the chronic, protracted nature of the human disease. This study did not assess the long-term efficacy and safety of the drug. Moreover, although multiple co-localization experiments strongly suggest a direct action of Nerandomilast on B cells, establishing absolute cell autonomy will require future validation using B cell-specific PDE4B knockout mouse models. Furthermore, the sample size, while adequate for detecting major phenotypic changes, limits the detection of subtler effects and robust subgroup analyses. Given the aforementioned limitations, the findings of this study should be regarded as preclinical proof-of-concept, providing robust mechanistic hypotheses and preliminary efficacy evidence for the clinical trial of Nerandomilast in IIM-ILD. However, the final efficacy and safety must be confirmed in rigorously designed clinical studies.

## Conclusion

In conclusion, our study confirms that the PDE4B inhibitor Nerandomilast is a potential therapeutic candidate for IIM-ILD. In preclinical models, Nerandomilast alleviates muscle inflammation, pulmonary fibrosis, and pulmonary inflammation. Its therapeutic mechanism involves elevating intracellular cAMP levels in B cells, inhibiting abnormal B cell proliferation and plasma cell differentiation, and synergistically regulating multiple key signaling pathways such as NF-κB, PI3K-AKT, STAT3, and CREB. Future research will further validate its long-term efficacy in more complex models resembling human diseases and explore deeper mechanisms of its involvement in B-cell regulation to accelerate its translation into clinical applications.

## Data Availability

The datasets presented in this study can be found in online repositories. The names of the repository/repositories and accession number(s) can be found in the article/**Supplementary Material**.
