# Supplementary figures and images for: Nerandomilast attenuates idiopathic inflammatory myopathy-associated interstitial lung disease via inhibiting proliferation and differentiation of B cells
Source: Front Immunol. 2026 Feb 18;17:1771007. doi: 10.3389/fimmu.2026.1771007 (PMC12956678; doi:10.3389/fimmu.2026.1771007)

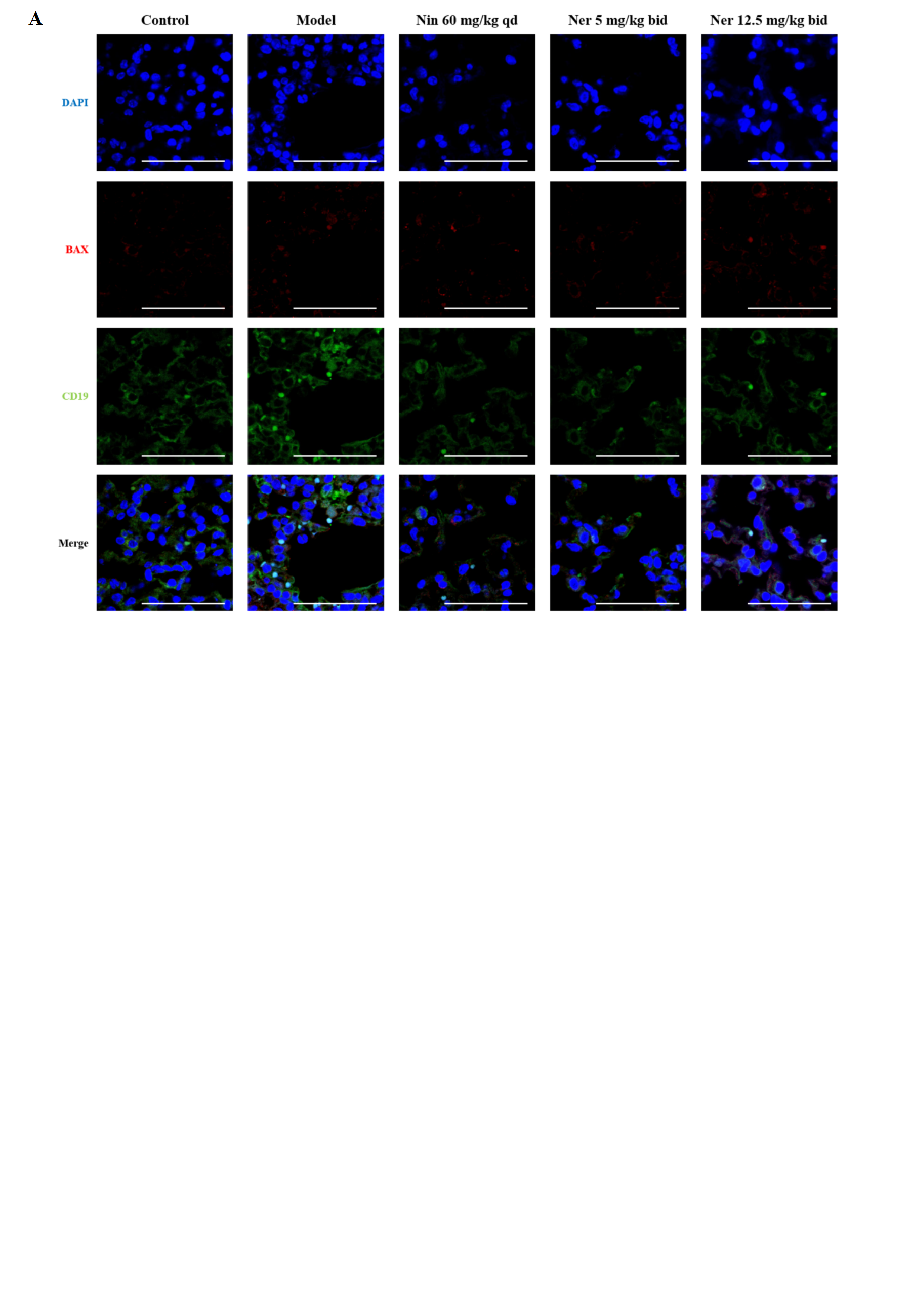

Supplement: Supplementary file 2 [file Image1.tif]
